# Preoperative simulated surgery on 3D model assists osteotomy feasibility verification and surgical guidance for patients with cubitus valgus/varus deformity: a retrospective observational study

**DOI:** 10.1186/s13018-023-03939-7

**Published:** 2023-06-29

**Authors:** Kai-Xiao Xue, Xing-Guo Zheng, Chang Qiao, Jia-Hu Fang

**Affiliations:** Department of Orthopedics, The First Affiliated Hospital of Nanjing Medical University, 300 Guangzhou Road, Nanjing, 210029 Jiangsu Province People’s Republic of China

**Keywords:** 3D printing, Simulated surgery, Osteotomy, Joint deformity, Elbow joint

## Abstract

**Background:**

As the common delayed complication of supracondylar fractures in children, cubitus valgus/varus deformity might lead to pain and loss of motion of the elbow. The current corrective treatment might not be accurate enough and even contribute to postoperative deformity. This study retrospectively analyzed the clinical value of preoperative simulated surgery on 3D model-assisted osteotomy feasibility verification and surgical guidance for cubitus valgus/varus deformity.

**Methods:**

Seventeen patients were selected from January 2017 and November 2019. Deformities were analyzed from imaging data and 3D models and corrected after the simulated operations. The radiographic evaluation comprised osseous union, carrying angle, and anteversion angle of the distal humerus. The clinical evaluation was performed according to the Hospital for Special Surgery (HSS) scoring system.

**Results:**

All patients underwent the operation successfully and had no postoperative deformity. The carrying angle was significantly improved postoperatively (*P* < 0*.*001). The anteversion angle of the distal humerus did not change significantly (*P* > 0*.*05). The HSS score rose after surgery (*P* < 0*.*001). The function of the elbow joint was excellent in seven cases and good in ten cases.

**Conclusion:**

Simulated surgery on 3D model plays an important role in osteotomy plan and surgical guidance, contributing to good surgical efficacy.

## Background

Elbow deformities, comprising cubitus varus and cubitus valgus, are one of the most common delayed complications of the distal humerus fractures in children, especially supracondylar fractures of the humerus [1–5]. While most of supracondylar fractures in children could be treated conservatively [6], such as cast immobilization and elevated, straight-arm traction, approximately 20% of supracondylar fractures require surgical treatment [7]. Without proper treatment, the incidence of cubitus varus after supracondylar fracture of the humerus is as high as 50% [8]. Compared with cubitus valgus, cubitus varus is more common [9].

Elbow deformities are often regarded as cosmetic problems rather than diseases in need of treatment. However, in addition to appearance, the deformities also bring about varying degrees of damage to the function of the elbow, such as limitations in the functional activities of extension and flexion, traumatic ulnar neuritis, joint pain, and instability [10]. In the clinic, when these degrees of damage occur, correction is necessary. Previous studies [11–13] have described different corrective supracondylar osteotomies as the standard treatment for elbow deformities: open/closed wedge osteotomy, French osteotomy, and dome osteotomy.

Whether accurate osteotomy can be performed strictly according to the preoperative steps planned can affect surgical outcomes. However, in the past, osteotomy performance has been mostly based on the clinical experience of orthopedic surgeons, and traditional deformity correction is often limited to the two-dimensional plane, which easily leads to postoperative deformity [14, 15]. Currently, with the wide use of three-dimensional printing techniques in orthopedics, there have been some related reports about the successful application of three-dimensional printing techniques in the clinical treatment of elbow deformities worldwide [16, 17].

In this study, we retrospectively analyzed the clinical effect of a three-dimensional printing technique applied to the treatment of complex elbow deformities in the orthopedics department of our hospital. The report is given in the following.

## Materials and methods

### Participants

Between January 2017 and November 2019, 17 patients with 18 elbow deformities (one patient had bilateral cubitus valgus), consisting of seven cubitus valgus and eleven cubitus varus deformities, underwent corrective osteotomy that adopted preoperative planning and simulated operations based on three-dimensional models. All patients were followed up for more than 12 months (range, 12–19 months; mean, 14.5 months). There were eight males and nine females, with a mean age of 26.4 years (range, 18–44 years) at the time of surgery. Our institutional review board approved this study. After informed consent was obtained from the patients for participation in the study, a preoperative plan and simulated operation based on three-dimensional models, corrective osteotomy according to the preoperative plan, and physical and radiographic examinations were carried out.

The inclusion criteria included a previous history of elbow fracture, a definite osseous deformity of the radius, ulna, or humerus, and an age of at least 18 years at the time of surgery, as well as complete follow-up data. The exclusion criteria included elbow deformity caused by osteochondrodysplasias and metabolic bone disease and elbow deformity caused by malunion of fracture after the age of 18 years.

In this study, all patients demonstrated angular deformities of the bone on plain radiographs. Surgery was indicated due to functional deficits or other complications, such as ulna neuritis, in all patients. Additionally, three of the patients complained about the appearance of their elbows. Other details are shown in Table 1.

**Table 1:** Baseline demographics and clinical preoperative characteristics

Case	Sex	Age at initial injury (y)	Duration of deformity (y)	Affected side	Category of deformity
1	M	14	23	R	Varus
2	F	10	10	R	Varus
3	F	25	9	L	Valgus
4	F	12	9	L	Varus
5	M	12	12	L	Varus
6	M	8	23	R	Varus
7	M	8	13	R	Varus
8	F	17	8	L	Varus
9	M	10	34	L	Valgus
10	F	12	7	L	Varus
11	F	8	11	L & R	Valgus
12	M	14	15	R	Valgus
13	F	13	19	L	Varus
14	M	12	6	R	Varus
15	M	8	28	R	Valgus
16	F	15	15	L	Varus
17	F	15	17	R	Valgus
Mean	F, 9; M, 8	12.5	15.2	R, 10; L, 8	Varus, 11; valgus, 7

### Preoperative procedure

#### Step 1: Simulation technique

**Fig. 1:**
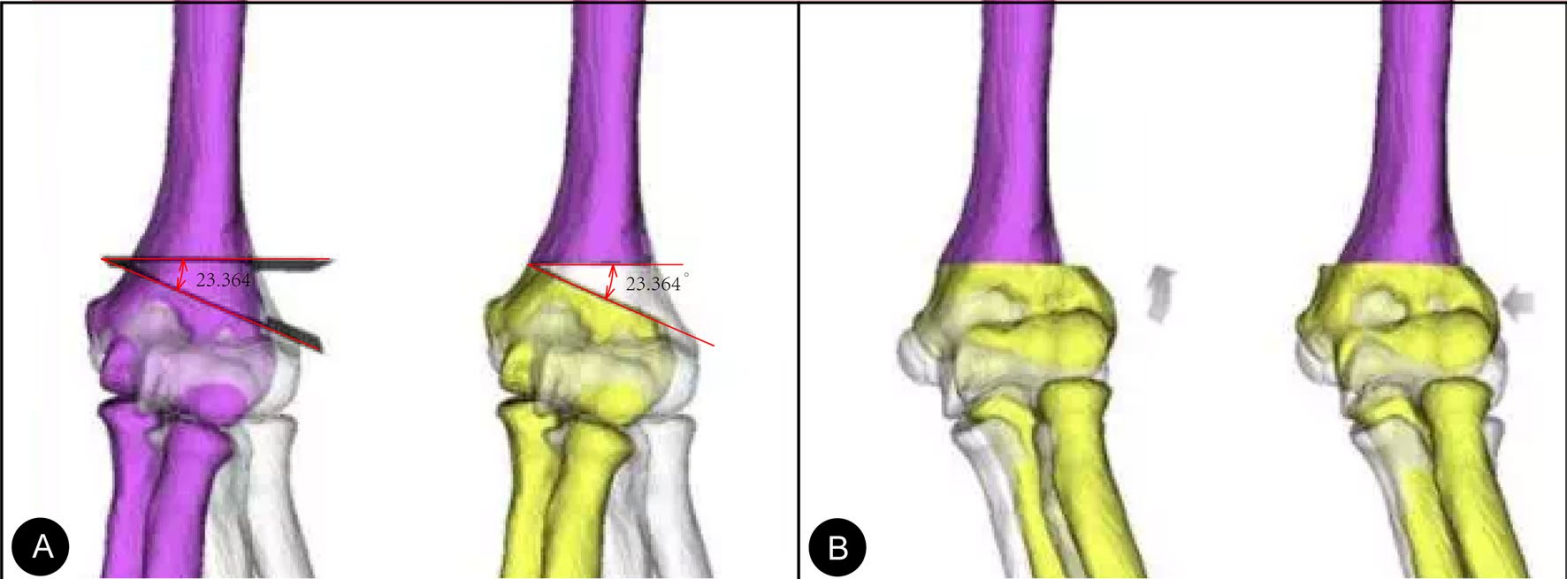
Simulation technique: **A** The flip model of the unaffected elbow joint was superimposed. **B** The ends of the bone were closed, and the correction was successful

Before surgery, multirow CT and digital X-ray imaging of the bilateral elbow joints were performed for every patient. Then, the CT data were imported into Mimics 21.0 (Materialise, Belgium) in DICOM format to construct 3D images of the bilateral elbow joints. On the computer, it was possible to construct a virtual model of the unaffected elbow joint (patients with bilateral elbow deformities used the normal elbow as a template for orthosis). The deformity of the affected bone was evaluated by superimposing the flip model of the unaffected elbow joint (Fig. 1A–B). This kind of model can determine the degree of deformity and the segments and planes of deformity. Then, on the premise of preserving the medial bone cortex (hinged bone cortex) as much as possible, preoperative simulated osteotomy was performed. After osteotomy, the gap was closed at an angle that allowed the carrying angle of the affected side to equal that of the unaffected side. This simulated angle was the correction angle in the subsequent actual surgery.

#### Step 2: 3D model preparation

After simulation, the data of the affected elbow joint were input into the 3D printer in STL format to construct a 3D model, which was built as 1:1 model by ABS (acrylonitrile butadiene styrene). SFF (solid freeform fabrication) technology was used [18].

#### Step 3: Simulated operation

**Fig. 2:**
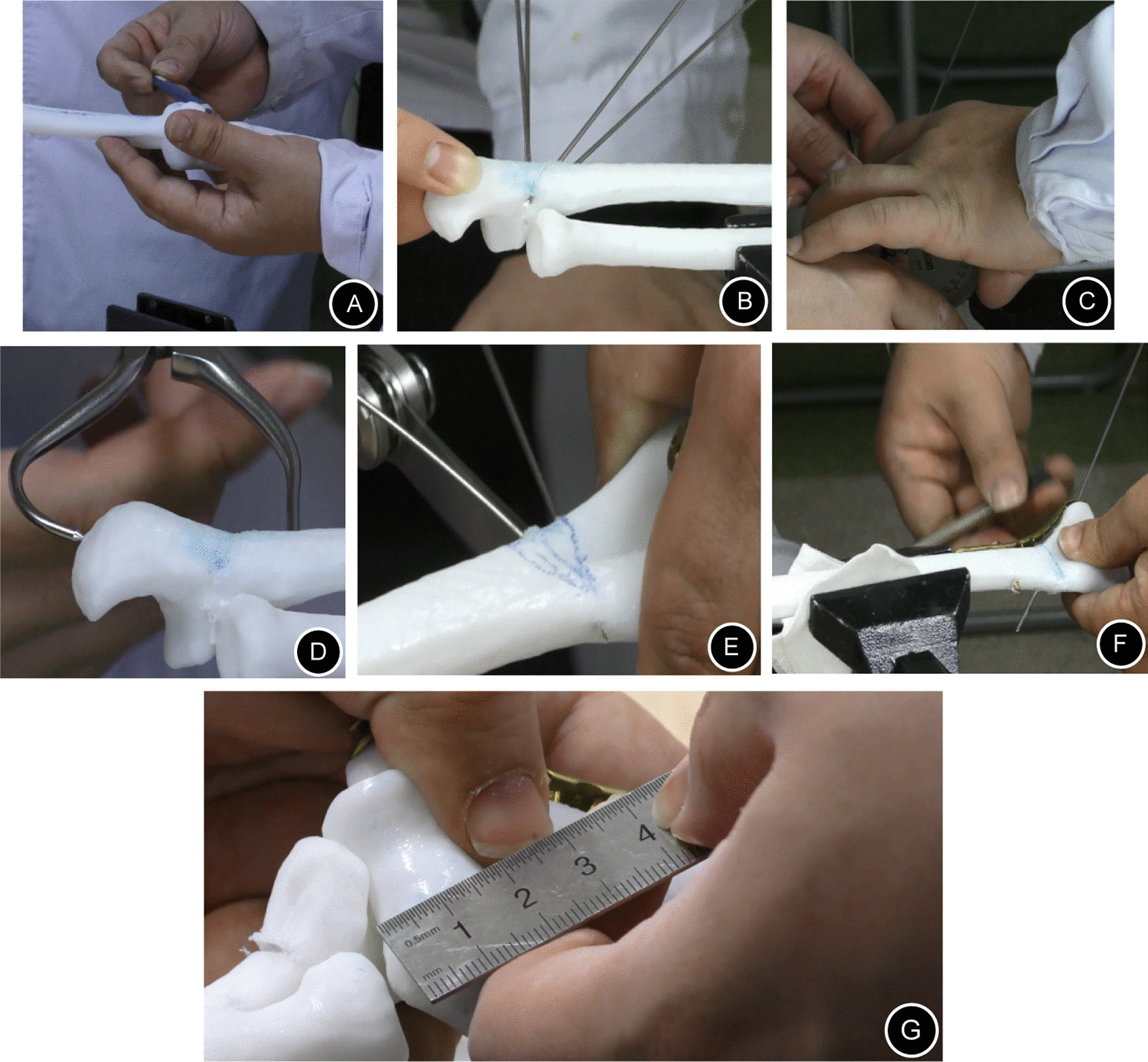
The preoperative simulated operation: **A**, **B**, **C** The deformity angle was measured, and the osteotomy level was determined. **D**, **E** Osteotomy of the proximal ulna and temporary fixation with point reduction forceps. **F** The conformed plate was fixed after preshaping. **G** The angle was measured again after orthopedic surgery

Before surgery, as with the preoperative evaluation for a normal deformity, different deformity angles needed to be measured in the coronal plane and sagittal plane, such as the carrying angle and ulnar deformity angle (if present) in the coronal plane, anteversion angle of the distal humerus in the sagittal plane, which were helpful for precisely calculating the deformity correction angle (Fig. 2A). Then, the related data were verified one by one on the 3D model on which these angles would be marked, and an appropriate osteotomy level was chosen in the humerus and/or ulna to perform closed wedge osteotomy (Fig. 2B–E). After osteotomy, the internal fixation devices were chosen, shaped according to actual osteotomy shapes, and disinfected for use in the actual operations (Fig. 2F).

Finally, the angle was measured, the deformity was fixed, the orthopedic operation was successfully completed, and the key steps and correction angles in the simulated operation were recorded as a reference for the actual operation (Fig. 2G).

### Intraoperative procedure

The patients were placed in the supine position under general anesthesia. A sterile tourniquet was applied. A posterior median incision was made in the elbow joint. Then, the skin, subcutaneous tissue, and deep fascia were cut open in turn. After exposure, the ulnar nerve was released and protected.

**Fig. 3:**
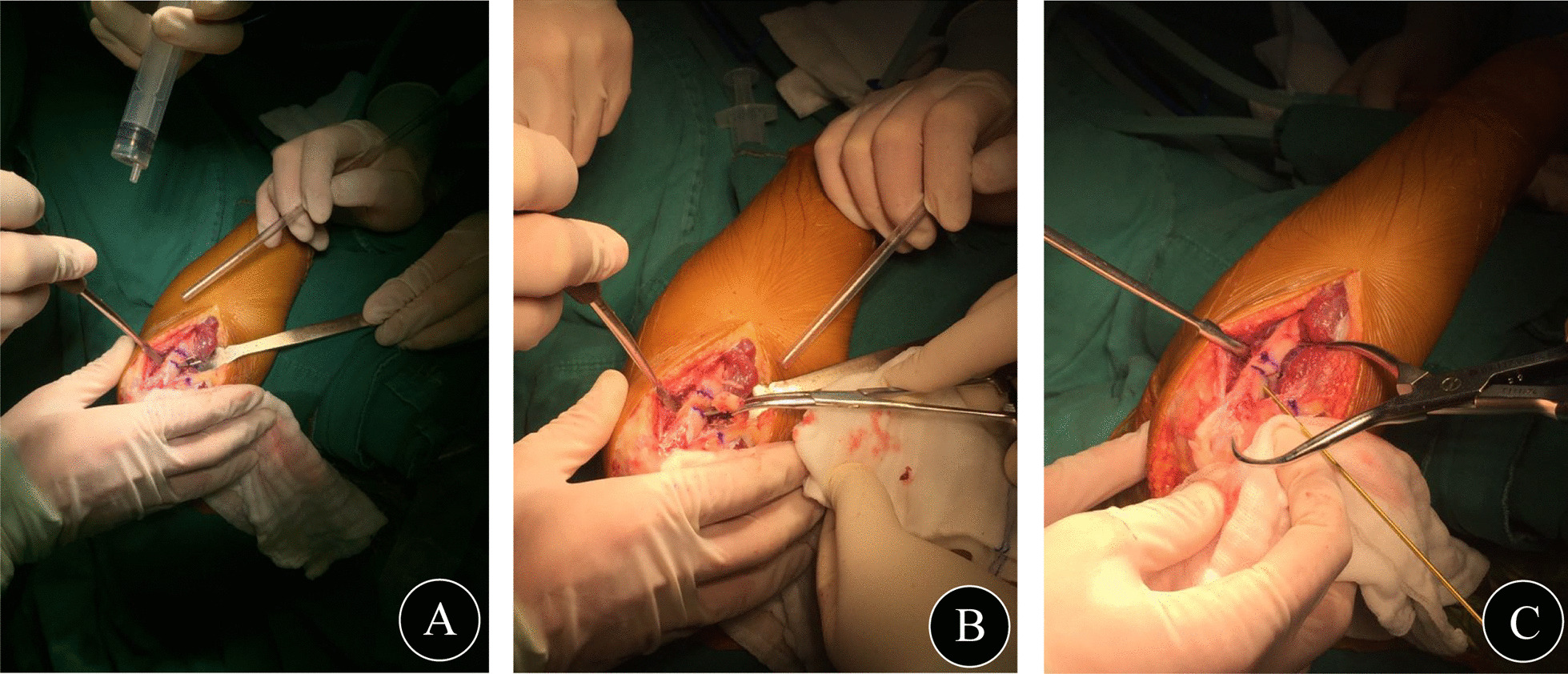
Closed wedge osteotomy of the proximal ulna in one patient: **A** Osteotomy of the proximal ulna was performed. **B** The wedge-shaped bone was removed by vascular forceps. **C** The osteotomy site was closed and temporarily fixed

According to the plan from the preoperative simulated operation, osteotomy of the elbow joint (distal humerus or/and proximal ulna) was performed with determined correction angles at the determined osteotomy level (Fig. 3A). First, the position and direction of the bone cuts were marked with K-wires using fluoroscopy. Then, osteotomy was performed with an oscillating saw following the placed K-wires. The wedge-shaped bone mass was removed by vascular forceps (Fig. 3B). After closing the bone gap, the alignment of the upper limbs was readjusted until the result was satisfactory (Fig. 3C). All osteotomies were fixed with preoperatively shaped, locked, and suitable plates. During surgery, the key steps and key data were recorded.

### Radiographic and clinical evaluation

Before surgery and at the most follow-up evaluation, radiographic and clinical evaluations were conducted for all the patients. The disappearance of the osteotomy line and continuity of the trabecular bone indicated complete union. For all the patients, the carrying angle, humeral deformity angle (defined by the longitudinal humeral axis and the upper ulnar and radial articular surface lines), and ulnar deformity angle (if present) (defined by the ulnar surface line and the upper ulnar and radial articular surface lines) in the coronal plane, as well as the anteversion angle of the distal humerus in the sagittal plane, were measured on plain X-ray to make orthopedic plans.

During the follow-up, the carrying angle and anteversion angle of the distal humerus were measured on plain X-ray and used to evaluate the elbow. To better evaluate the carrying angle, the valgus direction was considered positive, and the valgus direction was considered negative. The ranges of motion of the elbow were given attention. The HSS (Hospital for Special Surgery) score [19] was used to evaluate preoperative and postoperative elbow function.

### Statistical analysis

*SPSS* for Windows software (version 26.0, IBM^®^, Chicago, IL, USA) was used for statistical analysis. Statistical significance was set at *P* < 0.05. The Shapiro‒Wilk test was used to test the normality of the measurement data. Measurement data following a normal distribution are expressed as $\overline{x }$±*s*, and those with a nonnormal distribution are expressed as *M(Q*_25_–*Q*_75_*)*. For preoperative and postoperative data, as well as postoperative and simulation-operative data, a paired sample *t*-test was used when the data followed a normal distribution, and the Frank Wilcoxon test was used when the data did not conform to a normal distribution.

## Results

All osteotomy sites showed union after an average of approximately 3 months after surgery. For all patients, the carrying angle of the affected side was corrected by at least 12.5°. The average carrying angle of cubitus varus patients was 4.93° (varus alignment) before surgery and 14.04° (valgus alignment) after surgery, while that of cubitus valgus patients was 36.74° (valgus alignment) before surgery and 13.89° (valgus alignment) after surgery. These differences were significant (*P* < 0*.*001). In addition, the anteversion angle of the distal humerus in the sagittal plane showed little variation (40.48 ± 4.07 vs. 40.87 ± 2.90). This difference was insignificant (*P* > 0*.*05). Surgeries were done following the former simulated operations, and there was no significant difference in the data between actual operations and simulated operations (*P* > 0*.*05). All patients’ deformity in the coronal plane was successfully corrected, and all patients had no new deformity in the sagittal plane.

**Fig. 4:**
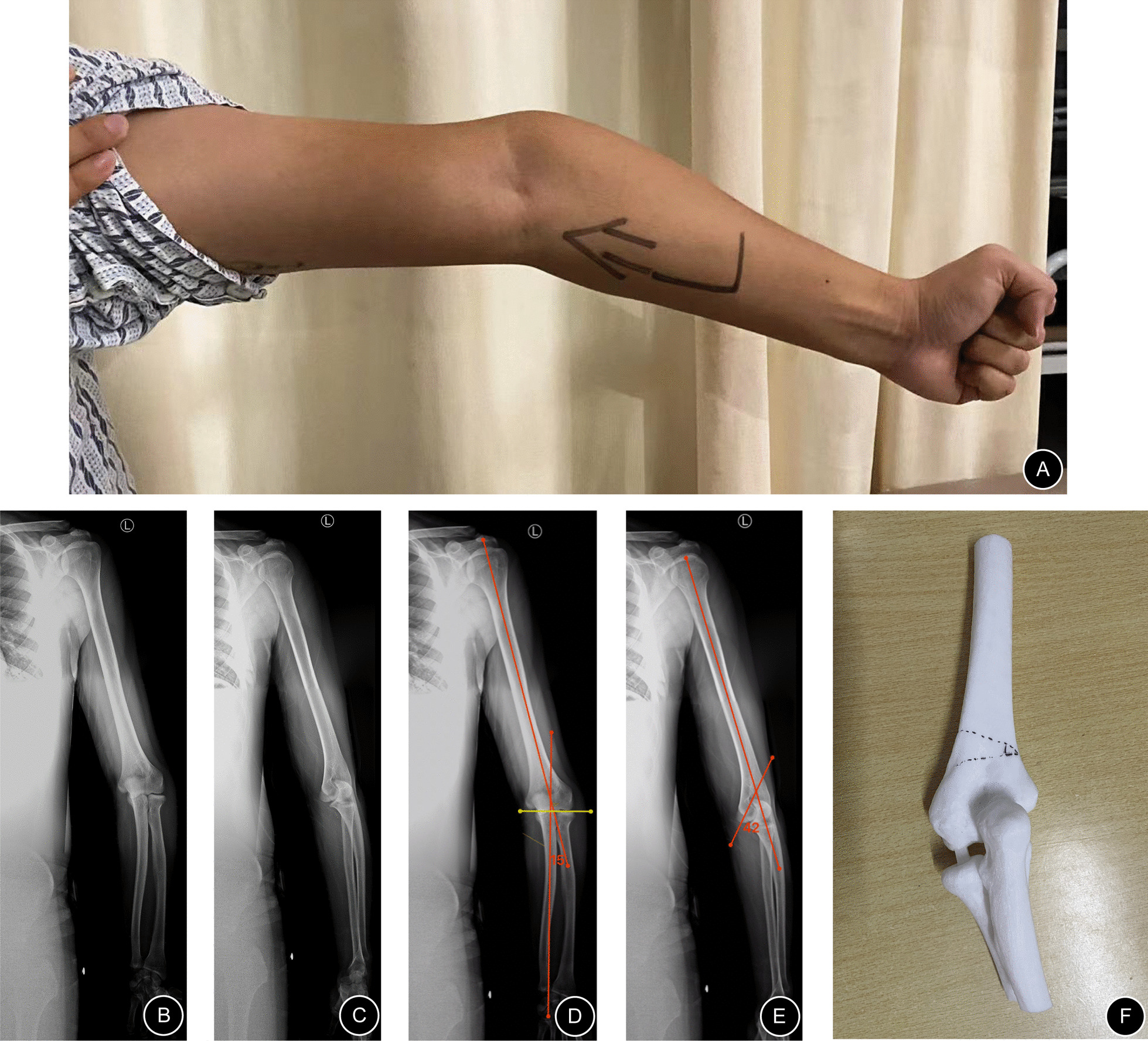
A 24-year-old man presented with a left cubitus varus deformity (Case 5). His chief complaint was mild pain with limited motion of the elbow: **A** Preoperative appearance. **B**, **C** Preoperative anteroposterior and lateral radiographs. **D**, **E** Preoperative deformity analyses show varus of approximately 15° and anteversion angle of the distal humerus of approximately 42°. **F** Osteotomy plan with the help of a 3D model

In one patient with bilateral cubitus valgus of the elbows (Case 11), given her complex deformity (Fig. 4), although the deformity was corrected successfully, the range of flexion and extension of her elbow joints after surgery was reduced compared with that when she was healthy (left, 20°–120°; right, 20°–130°). Except for Case 11, the range of flexion and extension of the elbow joint in the other patients did not change significantly. At the last follow-up, all the patients’ pain was eased, and varus/valgus appearances were improved. Other details are as follows (Tables 2, 3).

**Table 2:** Measurement of the relevant angles

	Carrying angle (°)			Anteversion angle of the distal humerus (°)		
	Pre-op	Simulated op	Post-op	Pre-op	Simulated op	Post-op
1	− 9.83	14.31	14.47	41.10	40.63	41.52
2	− 5.64	14.97	14.37	36.78	37.47	38.97
3	28.32	16.23	15.03	35.62	36.54	45.14
4	5.51	17.59	18.22	46.05	40.52	38.79
5	− 15.41	10.63	11.01	42.59	44.24	45.11
6	− 3.85	10.99	13.67	43.88	42.79	40.61
7	2.37	17.24	16.81	41.67	42.01	43.87
8	− 20.18	15.21	12.87	35.08	37.67	39.65
9	35.08	17.25	16.72	40.95	40.06	36.24
10	1.77	17.26	15.39	39.61	38.47	37.29
11	45.86/32.28	16.54/16.91	17.8/15.8	41.92/40.37	38.54/42.67	38.31/43.62
12	40.57	11.04	10.06	38.71	39.49	42.76
13	− 10.69	9.59	10.33	47.63	48.67	44.20
14	1.06	12.87	13.57	38.67	39.24	41.27
15	44.41	10.63	9.69	34.68	36.54	39.01
16	0.65	14.92	13.70	47.57	41.53	42.67
17	30.66	13.54	12.12	35.80	36.33	36.65
*SUM*						
Varus	− 4.93 ± 8.22	14.14 ± 2.80*	14.04 ± 2.28^†§^	40.48 ± 4.07	40.19 ± 3.15	40.87 ± 2.90^‡§^
Valgus	36.74 ± 6.92	14.59 ± 2.84*	13.89 ± 3.26^†§^			

*The difference in the data before surgery and during simulated surgery was significant (*P* < 0.001); ^†^The difference in the data before surgery and after surgery was significant (*P* < 0.001); ^‡^The difference in the data before surgery and after surgery was insignificant (*P* > 0.05); ^§^The difference in the data during simulated surgery and after surgery was insignificant (*P* > 0.05)

**Table 3:** Clinical evaluation of all the patients

Case	Preoperation	Postoperation	Z/t	*P* value
Pain	15.0 (15.0–20.0)	27.5 (25.0–30.0)	− 3.703	< 0.001
Function	14.0 (12.0–16.0)	19.0 (18.0–20.0)	− 3.655	< 0.001
Functional range in the sagittal plane	14.0 (13.0–15.0)	15.0 (14.0–16.0)	− 2.831	0.005
Muscle strength	8.0 (5.0–10.0)	10.0 (8.0–10.0)	− 2.549	0.011
Flexion contracture	4.0 (4.0–6.0)	6.0 (4.0–6.0)	− 1.000	0.317
Extension contracture	4.0 (4.0–6.0)	6.0 (4.0–6.0)	− 1.000	0.317
Pronation	4.0 (3.0–4.0)	4.0 (4.0–4.0)	− 1.633	0.102
Supination	3.5 (3.0–4.0)	4.0 (3.0–4.0)	− 1.342	0.180
HSS score	69.5 ± 6.2	88.7 ± 3.5	− 15.120	< 0.001
Excellent rate*	0	41.17%	–	–

*HSS scores of all patients were below good before surgery and were good or better after surgery

## Discussion

Cubitus varus and valgus are typical deformities of the upper extremity. They are often the delayed complications of supracondylar fractures in children. Symptoms and functional impairments follow and may cause serious disabilities [15, 20, 21]. Previously reported studies [22, 23] have shown that with the existence and progress of the deformity, the instability of the ligament will progress and eventually damage the ulnar nerve, causing various complications. Therefore, elbow deformity is a disease in need of treatment and not only a cosmetic problem.

Normally, elbow deformities result from the distal humerus in the coronal plane, and the treatment is supracondylar osteotomy of the humerus. According to related reported studies [11–13], there are many methods of supracondylar osteotomy of the humerus, including open/closed wedge osteotomy, French osteotomy, and dome osteotomy, among which wedge osteotomy is the most widely used. After performing a supracondylar osteotomy of the humerus, the patients’ elbow deformities improved immediately, and the elbow joint returned to a normal valgus angle (average angle of 12.88° ± 5.92) [24].

However, elbow deformities on a single plane account for a small percentage of cases [25]. Such deformities often involve multiple planes or even multiple segments, and the deformities are often not limited to the distal humerus, which is often a difficulty encountered by orthopedic surgeons in the clinic. To determine their real location, the deformities should be carefully analyzed to further evaluate the complexity of the deformity and determine the CORA (the center of rotation of angulation) point.

This requires deformity correction surgery to be more accurate and individualized. With the help of preoperative two-dimensional radiographic data, orthopedic surgeons need to seek new solutions to create more accurate orthopedic plans and perform more accurate osteotomies. The accuracy of the osteotomy plan should be verified before the actual surgery.

Fortunately, most of these needs can be met by three-dimensional printing techniques in orthopedics. Some previously reported orthopedic studies [26–28] have proven that visual and tactile feedback provided by 3D solid models help and support further understanding and analysis of the anatomy. With the assistance of these models, orthopedic surgeons are able to determine the osteotomy level and perform osteotomy more accurately [29]. A simulated operation can be performed on a 3D model and is beneficial for verifying post-orthopedic treatment in advance so that surgeons can adjust the operation plan at any time.

To resolve complex deformities accurately, we applied a 3D model to correct 17 cases of deformity, and we selected preoperative deformity analysis and simulated operations to guide actual deformity corrections. We did not select a navigation template because further exposure of the soft tissue at the elbow would make vascular and nerve injury more likely. We succeeded in correcting the different degrees of deformities in 17 cases. For simple elbow deformities, such as deformities of a single level or single plane, experienced orthopedic surgeons can analyze where the deformity and CORA truly are and plan the correct osteotomy level for surgery with the help of preoperative 2D radiographic data. Nonetheless, the benefits of the 3D model are that surgeons can rehearse the operation in advance to reduce intraoperative risks. Surgeons can also preshape steel plates to fit each patient’s specific bony anatomical bow.

For example, the patient in Case 5, a 24-year-old man, presented with a left cubitus varus deformity (Fig. 4). He presented with an obvious cubitus varus appearance (Fig. 4A). From preoperative anteroposterior and lateral radiographs (Fig. 4B–C), his forearm was more varus than that of a normal person. Additionally, the articular surface of the distal humerus matched well with the articular surface of the upper ulna and radius. We could easily comprehend that his deformity only comprised the deformity of the distal humerus in the coronal plane because his carrying angle was about − 15° (abnormal) and anteversion angle of distal humerus was approximately 42° (normal) (Fig. 4D–E). A supracondylar osteotomy of the humerus with a corrective angle of approximately 26° was sufficient (Fig. 4F). Considering that distal humeral osteotomy is quite a common osteotomy, the intraoperative imaging data are not shown in this article. After surgery, this patient recovered well. Appropriate correction was achieved, as evident on the radiographs (Fig. 7A–B). His appearance became normal, and the range of flexion and extension of the elbow joint on the affected side was great (0°–150°) (Fig. 7D–E). This range would meet all of his activity needs. At the last follow-up, the radiographic and clinical evaluation results showed a carrying angle of 11.01°, an anteversion angle of the distal humerus of 45.11° (Table 2), and a HSS score of 98 (excellent).

**Fig. 5:**
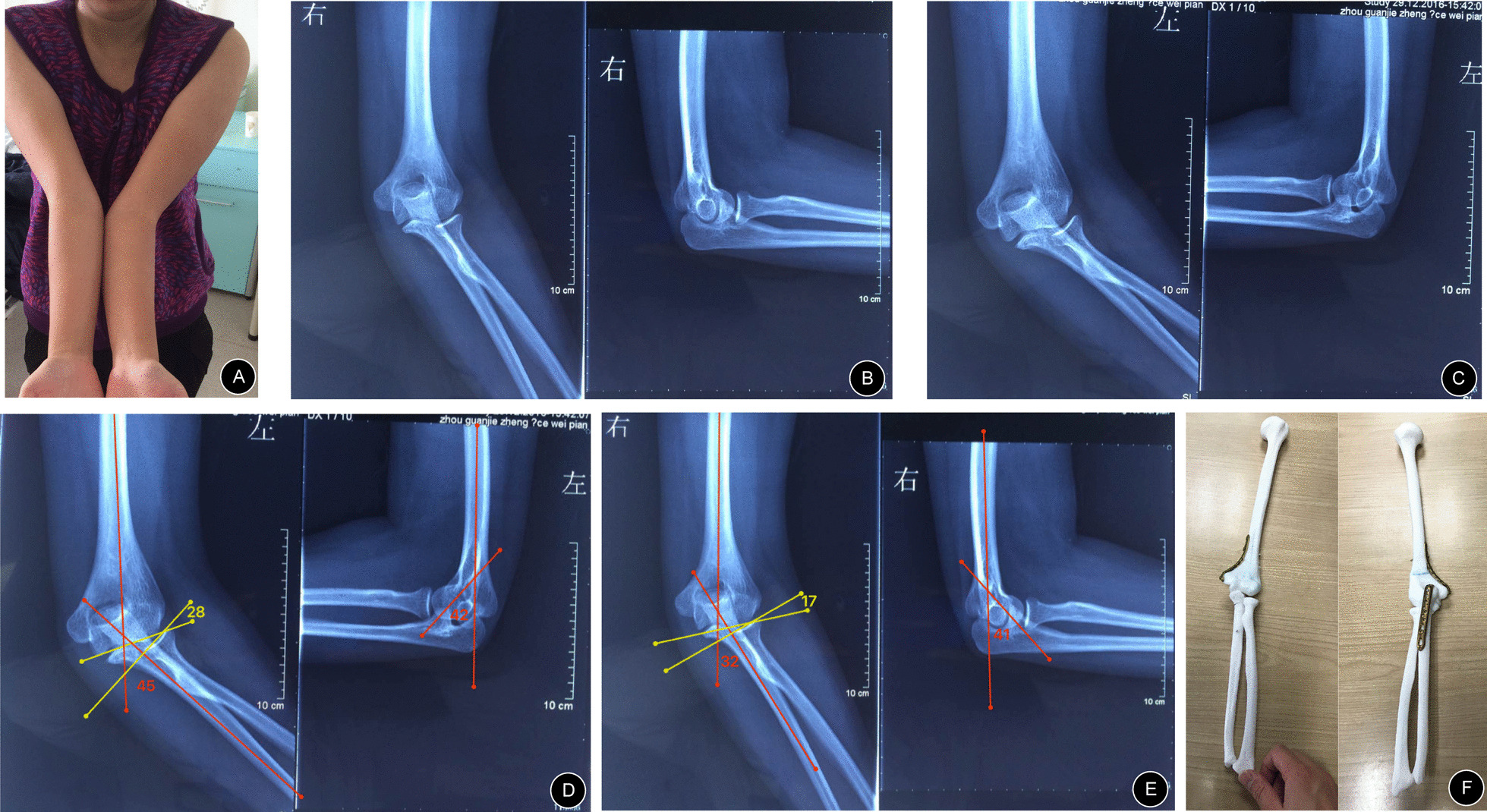
A 19-year-old woman presented with bilateral cubitus valgus deformities (Case 11). Her chief complaint was pain with limited motion of the elbow: **A** Preoperative appearance. **B**, **C** Preoperative anteroposterior and lateral radiographs. **D**, **E** Preoperative deformity analyses show valgus of approximately 45° and 32° in the elbow joints and deformities of approximately 28° and 17° in the proximal ulnae. In addition, the anteversion angles of the distal humerus were approximately 42° and 41°. **F** Osteotomy plan with the help of a 3D model

**Fig. 6:**
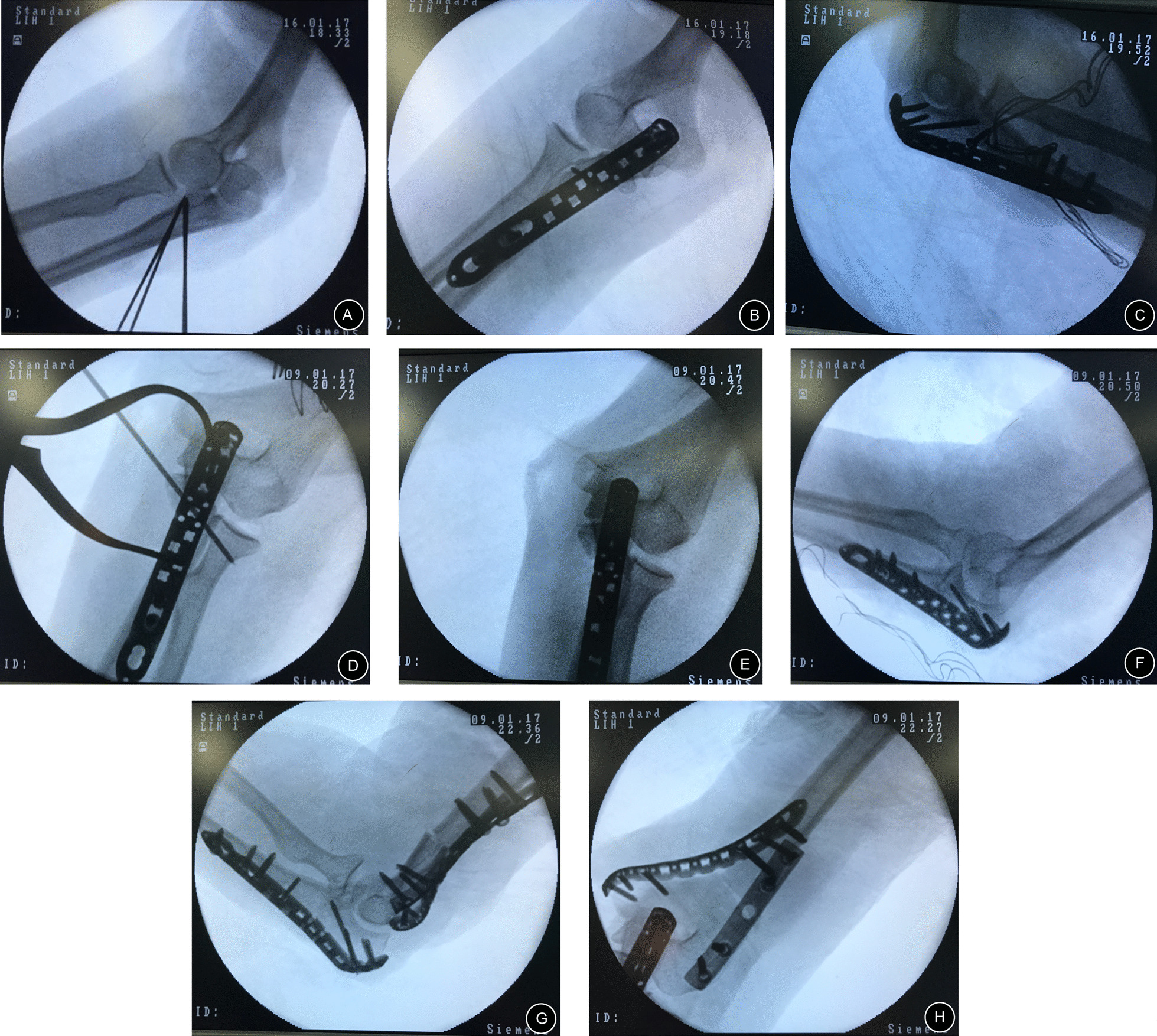
A 19-year-old woman presented with bilateral cubitus valgus deformities during surgery (Case 11): **A** Appropriate osteotomy correction of the right side was performed as in the simulated operation. **B**, **C** After the deformity of the right side was fixed, and the preshaped steel plate was prepared. **D** Appropriate osteotomy correction for the left side was performed as in the simulated operation. **E**, **F** After osteotomy of the proximal ulna, the deformity was examined. **G**, **F** Supracondylar osteotomy of the humerus continued to be performed, and the deformity was fixed. Preshaped steel plates were prepared

**Fig. 7:**
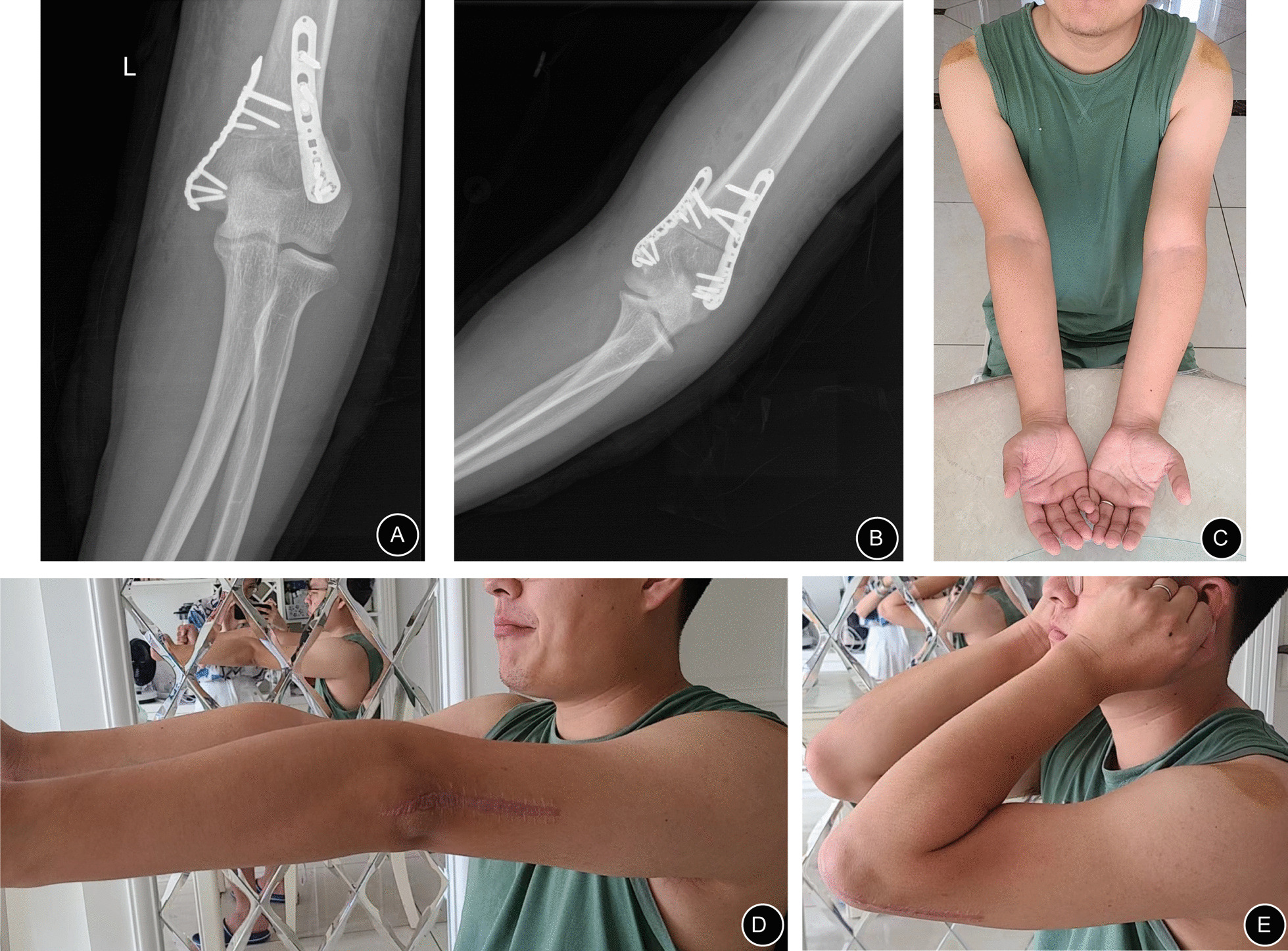
A 24-year-old man presented with a left cubitus varus deformity after surgery (Case 5): **A**, **B** Appropriate correction was achieved as evident on the postoperative anteroposterior and lateral radiographs. **C** The varus deformity was corrected. **D**, **E** The range of flexion and extension of the bilateral elbow joints after surgery remained unchanged (0°–150°)

**Fig. 8:**
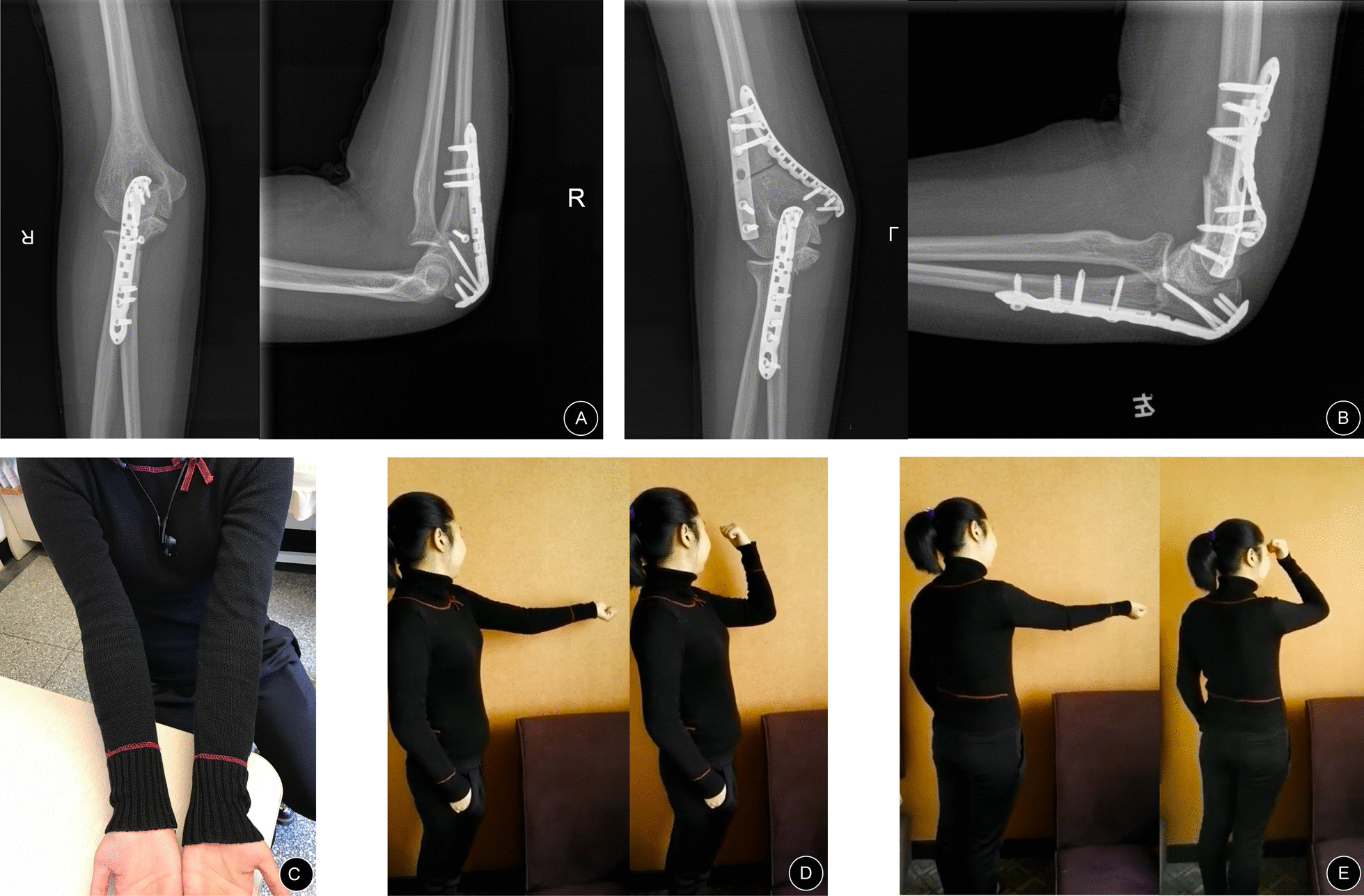
A 19-year-old woman presented with bilateral cubitus valgus deformities after surgery (Case 11): **A**, **B** Appropriate corrections were achieved as evident on the postoperative anteroposterior and lateral radiographs. **C** The valgus deformities were corrected. **D**, **E** The range of flexion and extension of the bilateral elbow joints after surgery was good (Left, 20°–120°; Right, 20°–130°)

In contrast, for complex deformities, such as deformities of different levels or different planes, simulated operations, in addition to the benefits mentioned above, can also help understand clinically rare deformities and verify ideas about preoperative plans and orthopedic procedures. We take an example, Case 11, a 19-year-old woman who presented with bilateral cubitus valgus deformities. The 3D model assisted greatly in the analysis of her complex deformity. Before surgery, when we analyzed her deformity from radiographs (Fig. 5B–C), we noticed that her elbows were severely valgus, and the bilateral articular surfaces of the distal humerus were not well matched with the bilateral articular surfaces of the upper ulna and radius. This was completely different from the patients with elbow deformities we have treated in the past. Based on the radiographs, we thought the left elbow deformity was limited to the coronal plane and comprised the deformities of the proximal ulna and distal humerus, and the right elbow deformity was limited to the coronal plane and comprised the deformity of the proximal ulna. To verify our analysis, we measured the deformities again on the 3D model and tried to perform osteotomy correction on the ulna and humerus of the 3D model. Our analyses of the deformities were correct, and we succeeded in correcting her deformities (Fig. 5F). The 3D model helped us select the proper osteotomy level in the ulna. We believe that osteotomy of the ulna should not only correct the ulnar deformity but also not affect the pronation and supination functions of the forearm after surgery. Additionally, the change in space between the humerus and radius after surgery and the matching degree of the upper ulnar and radial joints should be taken into consideration. Therefore, a simulated operation on the 3D model to verify our plan must be performed. Ultimately, we selected the correct osteotomy level in the proximal ulna during surgery (Fig. 6A), and the pronation and supination functions of the forearm after surgery were not affected during follow-ups (Fig. 7). The ranges of flexion and extension of the elbow joint were 20°–120° and 20°–130° (Fig. 8D–E), respectively. Although these ranges were not excellent, a previously reported study [30] showed that during daily life, a range of flexion and extension of the elbow joint of 30°–130° can meet 90% of the requirements of daily activities. Additionally, the patient was satisfied with the postoperative results. Therefore, the results were good. At the last follow-up, the radiographic and clinical evaluations revealed carrying angles of 17.8° and 15.8° and anteversion angles of the distal humerus of 38.31 and 43.62, respectively (Table 2), with HSS scores of 90 and 94 (excellent).

After correction, the patients’ anteversion angles of the distal humerus were somewhat affected by osteotomy. These changes did not have clinical significance (*P* > 0*.*05 from Table 2), and the average anteversion angle of the distal humerus was 40.87, which falls within the normal range, as some previously reported studies [31, 32] have shown.

## Conclusion

With the application of 3D models to perform simulated operations to guide orthopedic surgery, the correction effect and symptom improvement were obvious, especially for complex osteotomy or rare deformity (Tables 2, 3). The preliminary results of our seventeen patients indicate that this simulation technique is a clinically reliable method.

However, limited by technology, a previously reported study [33] indicated that there may be some differences between the 3D model and actual anatomy. The 3D model is an isolated model without soft tissue. Therefore, orthopedic surgery still needs to be performed by experienced orthopedic surgeons to avoid unnecessary risks.

The limitation of this study is that the sample size was small, and no control group was included. A larger sample size and more research are still needed to further evaluate the application value of 3D-printing technology in elbow orthopedics.

## Data Availability

The datasets used or analyzed during the current study are available from the corresponding author on reasonable request.
