# Correction: Preoperative simulated surgery on 3D model assists osteotomy feasibility verification and surgical guidance for patients with cubitus valgus/varus deformity: a retrospective observational study

**DOI:** 10.1186/s13018-023-04067-y

**Published:** 2023-08-08

**Authors:** Kai-Xiao Xue, Xing-Guo Zheng, Chang Qiao, Jia-Hu Fang

**Affiliations:** Department of Orthopedics, The First Affiliated Hospital of Nanjing Medical University, 300 Guangzhou Road, Nanjing, 210029 Jiangsu Province People’s Republic of China

**Correction: Journal of Orthopaedic Surgery and Research (2023) 18, 470** **https://doi.org/10.1186/s13018-023-03939-7**

Following publication of the original article [1], the authors identified an error in the abstract. The change is highlighted in bold typeface. “Seventeen patients were selected from October 2016 to November 2019” should be replaced with “Seventeen patients were selected from **January 2017** and November 2019”. “The original article [1] has been corrected”.
